# BrachyGuide: a brachytherapy‐dedicated DICOM RT viewer and interface to Monte Carlo simulation software

**DOI:** 10.1120/jacmp.v16i1.5136

**Published:** 2015-01-08

**Authors:** Evaggelos Pantelis, Vassiliki Peppa, Vasileios Lahanas, Eleftherios Pappas, Panagiotis Papagiannis

**Affiliations:** ^1^ Medical Physics Laboratory Medical School, University of Athens Athens Greece

**Keywords:** brachytherapy, dosimetry, Monte Carlo, treatment planning, QA

## Abstract

This work presents BrachyGuide, a brachytherapy‐dedicated software tool for the automatic preparation of input files for Monte Carlo simulation from treatment plans exported in DICOM RT format, and results of calculations performed for its benchmarking. Three plans were prepared using two computational models, the image series of a water sphere and a multicatheter breast brachytherapy patient, for each of two commercially available treatment planning systems: BrachyVision and Oncentra Brachy. One plan involved a single source dwell position of an ${}^{192}\text{Ir}$ HDR source (VS2000 or mHDR‐v2) at the center of the water sphere using the TG43 algorithm, and the other two corresponded to the TG43 and advanced dose calculation algorithm for the multicatheter breast brachytherapy patient. Monte Carlo input files were prepared using BrachyGuide and simulations were performed with MCNP v.6.1. For the TG43 patient plans, the Monte Carlo computational model was manually edited in the prepared input files to resemble TG43 dosimetry assumptions. Hence all DICOM RT dose exports were equivalent to corresponding simulation results and their comparison was used for benchmarking the use of BrachyGuide. Monte Carlo simulation results and corresponding DICOM RT dose exports agree within type A uncertainties in the majority of points in the computational models. Treatment planning system, algorithm, and source specific differences greater than type A uncertainties were also observed, but these were explained by treatment planning system‐related issues and other sources of type B uncertainty. These differences have to be taken into account in commissioning procedures of brachytherapy dosimetry algorithms. BrachyGuide is accurate and effective for use in the preparation of commissioning tests for new brachytherapy dosimetry algorithms as a user‐oriented commissioning tool and the expedition of retrospective patient cohort studies of dosimetry planning.

PACS numbers: 87.53.Bn, 87.53.Jw, 87.55.D‐, 87.55.Qr, 87.55.km, 87.55.K‐

## I. INTRODUCTION

In response to the increasing body of literature on the shortcomings of TG43‐based brachytherapy treatment planning systems (TPS),^1^, ^2^ advanced dose calculation algorithms beyond TG43 have been incorporated in commercially available TPSs. A grid‐based Boltzmann equation solver^3^, ^4^, ^5^ and a collapsed cone superposition algorithm^(6)^ can be used in BrachyVision and Oncentra Brachy, respectively, for patient specific dosimetry in HDR ${}^{192}\text{Ir}$ applications.

This paradigm shift renders the already acknowledged^(7)^ need for augmenting QA procedures imperative, since data to support commissioning procedures are lacking.^(8)^ Besides acceptance testing, such procedures are required to warrant that the global uniformity of brachytherapy practice that helped establish an improved standard of care and greatly facilitates interinstitutional trials is maintained in the transition from the robust and universally employed TG43 formalism to different dosimetry algorithms or algorithm implementations. TG186 has proposed a graded approach to the commissioning of TPSs employing advanced dose calculation algorithms, based on test cases and corresponding reference dosimetry data.^(8)^ Monte Carlo (MC) simulation is undoubtedly a valuable source for such reference data. MC methods can also be used in retrospective patient cohort studies to assess the impact of introducing advanced dose calculation algorithms for the treatment of specific brachytherapy sites. In both cases, simulations would have to be performed in the geometry defined through images available in DICOM format using information parsed from plans exported in DICOM RT format.

BrachyGuide is a brachytherapy dedicated software tool developed to expedite the fool‐proof configuration of input files for such MC simulations, featuring also a graphical user interface and a DICOM RT viewer. It is named after the acronym of a research project for the preparation of user oriented QA tools and the evaluation of advanced brachytherapy dosimetry algorithms in patient cohorts. It is not unique in the sense that similar software tools that are more comprehensive than the current version of BrachyGuide have been presented or announced in the literature.^9^, ^10^, ^11^ ALGEBRA,^(11)^ based on Geant4,^(12)^ and BrachyGUI, based on PTRAN_CT,^(13)^ have been successfully used for patient specific dosimetry in HDR gynecologic interstitial brachytherapy^(14)^ and endorectal brachytherapy,^(15)^ respectively. Still, BrachyGuide has its merits including availability^*^, focus on HDR ${}^{192}\text{Ir}$ brachytherapy dosimetry benchmarking, speed, and currently being the only DICOM RT interface to the widely used MCNP code^(16)^ for brachytherapy.

This work presents an overview of BrachyGuide functions, and results of calculations performed for its benchmarking. Indicative results of comparisons between treatment planning dosimetry employing advanced algorithms implemented in BrachyVision v.10.0.33, as well as Oncentra Brachy v.4.4, and corresponding MC simulations using BrachyGuide are also presented.

## II. MATERIALS AND METHODS

### A. The BrachyGuide software tool

**Figure 1:**
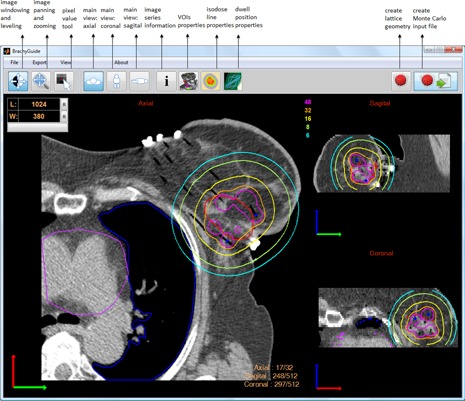
A print screen of the BrachyGuide interface with main function tools annotated.

BrachyGuide was developed using MATLAB (Math Works, Natick, MA). It is distributed as a compiled application executable on any machine with MATLAB Compiler Runtime installed. BrachyGuide parses and displays information from DICOM RT image, RT plan, RT structure set, and RT dose information object definitions (IOD). It features a user‐friendly graphical user interface (see Fig. 1) with several common capabilities such as: toggling between main and ancillary views, scroll and indexed image navigation, image window leveling, image pan and zoom, pixel value display, display and control of structures, source dwell positions, and isodose lines.

The main feature of BrachyGuide, however, is the automatic preparation of an MCNP input file from parsed information. MCNP is a choice reflecting nothing more than prior research experience, and other codes will be supported in future updates.

The computational model geometry for MC simulations is configured using the capability of the MCNP code to define rectangular lattice geometries. The size of each lattice element is equal to the voxel size of the imported X‐ray CT images, and the outer dimensions of the lattice geometry coincide with those of the CT image volume. Hence, the total number of lattice elements equals the number of voxels in the imported CT stack and the computational model geometry is identical to that available to the TPS, regardless of how the latter uses it. The user is also presented with an option to reduce memory requirements and simulation time by down‐sampling the in‐plane resolution of the imported CT images (by a factor of 2, 4, or 8) before MC input file generation.

The assignment of mass density, truncated to two significant digits, is performed on an individual voxel basis using either a default or a user‐defined CT calibration of Hounsfield units versus density. There is also an option to apply a density binning scheme over the 3D density cube of the computational model before MC input file generation in order to increase the efficiency of the corresponding simulation.^15^, ^17^, ^18^, ^19^ BrachyGuide uses an extended density binning function containing 54 density bins, which has been found to affect dosimetry less than 1% at distances up to 10 cm from ${}^{192}\text{Ir}$ sources for different human tissue elemental compositions.^(20)^ Mass density is used to assign elemental composition to each voxel using a look‐up table of 23 human tissue composition bins prepared from data in Schneider et al.^(21)^

Brachytherapy sources are represented in the generated input file by means of precalculated, source‐specific phase space files containing the energy, position, and direction of photons emerging from a source, for $8\times 10^{7}$ initially emitted photons. These are rather large files to distribute and a feasible alternative would be the distribution of the input file to generate them. A transformation must be applied to each photon position and direction read from the phase space file to account for source dwell position and the source orientation in it. BrachyGuide calculates and stores a transformation matrix for each dwell position of the treatment plan based on information retrieved from RT plan and RT structure set. Since RT plan includes only the source center coordinates in each of its planned positions, the corresponding source directions are obtained using the coordinates of the two catheter points closest to each dwell position. The transformation matrix to be applied for each photon read from a phase space file is sampled from a probability distribution calculated using the fraction of source dwell times to the total irradiation time. Since the source structure is not included in the input file generated by BrachyGuide, backscattered photons traversing the source volume are not considered correctly in the corresponding simulations. The dosimetric effect is expected to be slight due to the limited number of these photons in view of the small volume of HDR ${}^{192}\text{Ir}$ sources.

Absorbed dose is calculated using the F6 tally of the MCNP MC code, which scores the collisional kerma to medium in medium in the full computational model. BrachyGuide also includes a *FMESH4 tally in the input file to score photon energy fluence in each lattice element within the spatial extent of the DICOM RT dose grid, weighting the output by water mass energy absorption coefficients^(22)^ to obtain water kerma in medium in the computational model geometry. No variance reduction techniques are included in the MCNP input file generated by the current version of BrachyGuide. The current version of BrachyGuide also does not support applicators that can be included in the simulation geometry by manually editing the input file.

### B. TPS calculations

TPS calculations of this work were performed using two commercially available systems employing advanced dose calculation algorithms besides TG43 (BrachyVision v.10.0.33, Varian Medical Systems, Palo Alto, CA and Oncentra Brachy v.4.4, Nucletron, an Elekta company, Veenendaal, The Netherlands). Details of these algorithms and their implementation, which are beyond the scope of this work, can be found in the cited literature.^3^, ^4^, ^5^, ^6^, ^8^, ^23^, ^24^, ^25^, ^26^

Two computational models were used, a water sphere and a patient, to generate three plans with each TPS: TG43 water sphere, TG43 patient, and inhomogeneous patient.

For the TG43 water sphere plans, a mathematical model of a water sphere (15 cm radius) in air was converted to a series of DICOM X‐ray CT images (1 mm slice thickness, 340 mm FOV, 256 matrix). The images were imported to the TPSs and a plan was created consisting of one catheter with a single dwell position of a brachytherapy source at the center of the water sphere. The source was either VariSource VS2000^(27)^ (Varian Medical Systems) or microSelectron mHDR‐v2^(28)^ (Nucletron), depending on the TPS. The TG43‐based algorithms of the TPSs were used to calculate the dose around the source. The image, RT plan, RT structure set, and RT dose files were exported from the TPSs and imported to BrachyGuide to prepare corresponding MC input files. Since the water sphere test resembles the conditions assumed in the TG43 formalism, comparison of MC results with corresponding RT dose data, as well as published TG43 results, serves as a benchmark for BrachyGuide parsing of information from plans exported in DICOM RT format and the accuracy of phase space file usage.

For the TG43 patient plans, an actual plan of a multicatheter breast brachytherapy patient was used. The plan was originally performed in Oncentra Brachy with a dose prescription of 32 Gy at the periphery of the PTV. The TG43‐based algorithm of the TPS was used to calculate dose. Subsequently, the RT plan, RT structure set, and RT dose files were exported from the TPS. Before importing the DICOM RT files to BrachyGuide for the preparation of a corresponding MC input file, the patient CT images were modified by setting HU=0 (equal to that of water) for voxels lying within a 15 cm radius sphere around the centroid of the source dwell position distribution, and HU=‐1000 (equal to that of air) for all other voxels. Hence, the computational model geometry in MC simulations resembles the conditions assumed in the TG43 formalism, and MC results can be compared to corresponding RT dose data for benchmarking BrachyGuide in multicatheter multisource applications.

Since DICOM RT information is not uniquely transferable between TPSs and in view of the different HDR source used in each system, a different plan was performed in BrachyVision for the same patient images, and the above process was repeated to obtain a corresponding MC input file via BrachyGuide.

For the inhomogeneous patient plans, dose calculations for the TG43 patient plans were performed using the grid‐based Boltzmann equation solver and collapsed cone superposition algorithms of the BrachyVision and Oncentra TPSs, hereafter to be referred to as Acuros and ACE (Advanced Collapsed cone Engine) results, respectively. The corresponding DICOM RT files were imported to BrachyGuide without any modification to prepare MC input files for calculations in the same inhomogeneous patient computational model geometry available to the TPSs.

### C. MC simulations

Six MC simulations were performed using version 6.1 of the MCNP general purpose MC code^(16)^ with input files prepared using BrachyGuide (one for each of the TG43 water sphere, TG43 patient, and inhomogeneous patient plans from each TPS).

No modification was made to the input files, except for those for the TG43 water sphere plans where the cylindrical source symmetry was exploited by replacing the F6 tally by its equivalent tmesh1 tally with the pedep option that overlays a cylindrical meshgrid (CMESH) over the simulation geometry.

Simulations were performed for a number of photons equal to the number of entries in the source phase space files used. Type A (statistical) uncertainty depends on geometry and position. Indicative results of the maximum corresponding relative error, as calculated by the code, are 3% in simulations for the TG43 water sphere plans and 6% at the central plane of simulations for the patient plans. MC type A uncertainty is expected to be the main influence to the comparison of corresponding MC and TG43 RT dose results for benchmarking BrachyGuide. This is because the ${}^{192}\text{Ir}$ spectrum used in this work for source phase space file calculations^(29)^ was the same as in the original studies for determining the TG43 data used in the TPSs,^27^, ^28^ and differences due to the use of EPDL97 cross‐section data in this work and Angelopoulos et al.^(27)^ and the use of DLC99 cross‐section data in Daskalov et al.,^(28)^ are expected to be small for the ${}^{192}\text{Ir}$ energies. The same is assumed to apply for the indicative comparisons of MC and corresponding Acuros and ACE results, although the ${}^{192}\text{Ir}$ photon spectrum used in the corresponding algorithms and the cross sections used in the latter are not known. Hence, differences greater than type A uncertainty in the comparisons presented in the following sections must be attributed to MC or TPS related sources of type B (nonstatistical) uncertainty, other than the above mentioned items.

MC results and corresponding RT dose results were compared using custom routines. For dose‐volume histogram (DVH) calculations, organ coordinates from the RT structure set files were used, except for skin that was not contoured in the planning sessions. Skin was defined via morphological image processing by subtracting the outcomes of a single pass erosion and dilation.

## III. RESULTS

### A. TG43 water sphere plans

**Figure 2:**
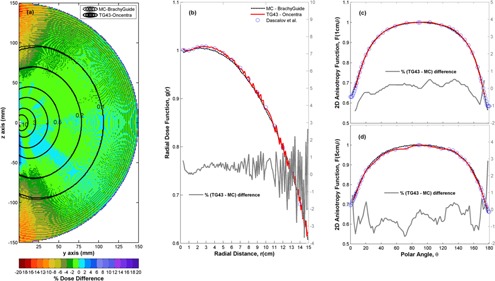
Comparison of Oncentra RT dose results and corresponding MC simulations using BrachyGuide for the TG43 water sphere plan, in terms of: (a) a color map representation of the spatial distribution of percentage dose differences ($100\%\frac{\text{TPS}‐\text{MC}}{\text{MC}}$) (b) radial dose function results, g(r), (c) 2D anisotropy function results, F(r=1cm,Θ), (d) 2D anisotropy function results, F(r=4cm,Θ). Corresponding MC results of Daskalov et al.^(28)^ are also presented for comparison in (b) through (d).

Oncentra Brachy RT dose results and corresponding MC simulations using BrachyGuide for the TG43 water sphere plan are compared in Fig. 2. Isodose lines and the color map of percentage differences presented in Fig. 2(a) show that MC and TPS results agree within ±2% for the vast majority of points around the mHDR‐v2 source. Noticeable differences are observed at points lying away from the source and close to its longitudinal axis, where MC results are greater by up to 16%. It is also worth noting the pattern of percentage differences that is discerned in the color map of Fig. 2(a) at distances greater than 5 cm from the source center. This is probably attributed to the interpolations and extrapolations of data from Daskalov et al.^(28)^ by the TPS, as also supported from the form of TPS data in Fig. 2(b). In this figure, g(r) results calculated using the line source approximation from MC and TPS RT dose data agree within ±1% at distances less than 5 cm, and ±3% close to the edges of the computational model. Corresponding differences of MC data of this work and Daskalov et al.^(28)^ in the data grid of the latter are under 1%.

In Figs. 2(c) and 2(d), F(r,Θ) values calculated from MC and TPS RT dose data agree within ±1.5%, except for angles close to the source cable (Θ=180°) at r=5cm where differences close to 3% can be observed. Corresponding differences of MC data of this work and Daskalov et al.^(28)^ for r=1cm and 5 cm are under 1.5%, except for r=5cm and Θ=178° where they reach 2.4%.

**Figure 3:**
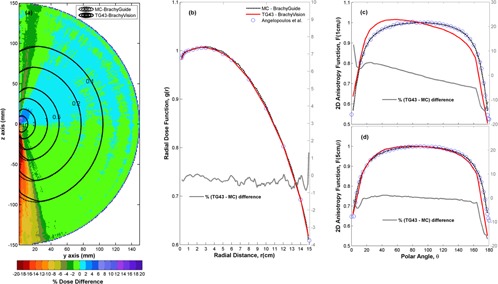
Comparison of BrachyVision RT dose results and corresponding MC simulations using BrachyGuide for the TG43 water sphere plan, in terms of: (a) a color map representation of the spatial distribution of percentage dose differences ($100\%\frac{\text{TPS}‐\text{MC}}{\text{MC}}$) (b) radial dose function results, g(r), (c) 2D anisotropy function results, F(r=1cm,Θ), (d) 2D anisotropy function results, F(r=5cm,Θ). Corresponding MC results of Angelopoulos et al.^(27)^ are also presented for comparison in (b) through (d).

Figure 3 summarizes the comparison of BrachyVision RT dose results and corresponding MC simulations using BrachyGuide for the TG43 water sphere plan with the VS2000 source. In Fig. 3(a) it can be seen that MC results are in close agreement with TPS results (±2%), except for points close to the source tip and its longitudinal axis. The significantly higher MC values close to the source cable (z<0) are explained by a difference in the cable length assumed in the source model employed for simulations of this work for phase space calculations, and in Angelopoulos et al.^(27)^ for the generation of TG43 data included in the TPS. Only 0.3 cm of cable was assumed in this work to resemble BrachyVision Acuros algorithm assumptions^(3)^ relative to 15 cm assumed in the Angelopoulos study. The remaining differences are attributed to the TPS. As shown in Fig. 3(b), g(r) results calculated using the line source approximation from MC and TPS RT dose data agree within each other, as well as data in the Angelopoulos study, within ±1%. A corresponding good agreement is observed in Figs. 3(c) and 3(d) between F(r,Θ) results of MC calculations of this work and Angelopoulos et al.,^(27)^ except for points close to Θ=180° for reasons explained above. TPS results in Figs. 3(c) and 3(d), however, present an abnormal trend and exhibit significant differences from both the other two datasets in these figures, which explain the differences observed in Fig. 3(a) in the source tip side (z>0). This abnormal trend of TPS TG43 data has been reported in previous studies in the literature.^(23)^

### B. TG43 patient and inhomogeneous patient plans

**Figure 4:**
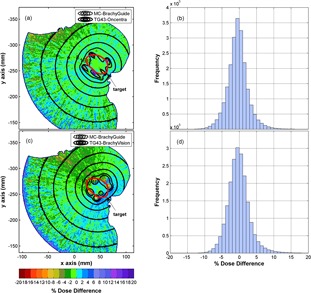
Comparison of Oncentra and BrachyVision RT dose results and corresponding MC simulations using BrachyGuide for the TG43 patient plans in terms of: (a) and (c) color map representations of the spatial distribution of percentage dose differences ($100\%\frac{\text{TPS}‐\text{MC}}{\text{MC}}$) in the central axial CT slice, with selected MC and TPS isodose lines superimposed, and (b) and (d) frequency histograms of percentage dose differences as in (a) and (c) in the whole computational model.

Oncentra and BrachyVision RT dose results are compared to corresponding MC simulations using BrachyGuide for the TG43 patient plans in Fig. 4. It should be noted that points of the computational model (configured as described in Material & Methods section B) laying outside the patient contour or corresponding to air are excluded from comparisons in Fig. 4.

The agreement of MC is excellent with both TPSs, as shown by the isodose lines and color maps presented in Figs. 4(a) and 4(c). Differences are within ±3% at the vast majority of points as confirmed also by the dose difference histograms presented in Figs. 4(b) and 4(d). Differences beyond MC type A uncertainty are observed close to the source dwell positions. These can be attributed to TPS extrapolations, as well as the fact that voxels inside the catheters were not excluded from comparisons. Differences between the two TPSs and corresponding MC results in the TG43 water sphere plans (see Figs. 2 and 3), are abated in Fig. 4 by the multiplicity of source dwell positions. A TPS dose underestimation relative to MC reaching ‐10% can be observed only at a limited number of points in the source tip direction (top left quarter in Figs. 4(a) and 4(c)) at increased distance from the implants. The dose overestimation of BrachyVision TG43 results in the periphery of the computational model in Fig. 4(c) (in the negative x axis side) is explained by the failure of the computational model to resemble the conditions assumed in the TG43 formalism at these points. The majority of the source dwell positions close to the presented CT slice lie closer to the periphery of the model than the centroid of their distribution (see Material & Methods section B) leading to the typical pattern of scatter dose overestimation from TG43 algorithms at points close to geometry boundaries and away from the implant.^(30)^

**Figure 5:**
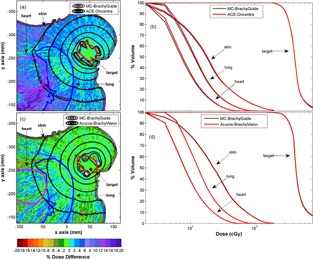
Comparison of Oncentra ACE and BrachyVision Acuros RT dose results and corresponding MC simulations using BrachyGuide for the inhomogeneous patient plans in terms of: (a) and (c) color map representations of the spatial distribution of percentage dose differences ($100\%\frac{\text{TPS}‐\text{MC}}{\text{MC}}$) in the central axial CT slice, with selected MC and TPS isodose lines superimposed, and (b) and (d) DVH data relative frequency histograms of percentage dose differences in the computational model.

Indicative results of comparisons between Oncentra ACE and BrachyVision Acuros RT dose results and corresponding MC simulations using BrachyGuide for the inhomogeneous patient plans are presented in Fig. 5. Following the convention of the TPS versions used in this work, MC results correspond to medium kerma in medium in Figs. 5(a) and (b) and water kerma in medium in Figs. 5(c) and (d). A later version of BrachyVision includes the option to calculate medium dose in medium to comply with TG186 recommendations^(8)^ even though tissue density is the determining factor for individualized patient dosimetry for the ${}^{192}\text{Ir}$ energies and, apart from density, all human tissues (except for bone) can be considered almost water equivalent.^(20)^

**Figure 6:**
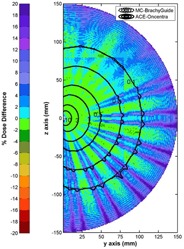
Comparison of Oncentra ACE RT dose results and corresponding MC simulations using BrachyGuide for the TG43 water sphere plan, in terms of a color map representation of the spatial distribution of percentage dose differences ($100\%\frac{\text{TPS}‐\text{MC}}{\text{MC}}$).

The findings of the comparison between Acuros and corresponding MC results in Figs. 5(c) and (d) are similar to corresponding findings presented in the literature^(5)^ without the use of BrachyGuide, thus serving as an indirect verification of its accuracy in the preparation of the MC input files. In Figs. 5(a) and 5(b), an excellent agreement can be observed between Oncentra ACE and MC results for the PTV and points located close to it. At points at relatively increased distances from the implant ACE can be seen to overestimate dose. This is due to the algorithm calculation settings that are preconfigured to optimize computation speed. As shown in Fig. 6, where ACE results are compared to corresponding MC calculations for the water sphere plan, as distance from the source increases beyond a few cm that is considered the primary range of interest to brachytherapy, ray effects become evident and the ACE algorithm tends to overestimate dose. The switch of resolution of the multiresolution Cartesian calculation grid employed by the algorithm is also discerned at 8 cm.

## IV. DISCUSSION

Comparisons of MC calculations to corresponding TPS results show that, besides being fast and effortless, BrachyGuide is also an accurate way of preparing MCNP input files for brachytherapy plans exported from TPS in DICOM RT format. Differences greater than MC type A uncertainties were explained by TPS‐related and other sources of type B uncertainty. These differences, presented in Figs. 2, 3, and 6 for single‐source dwell position comparisons, are abated in clinical plans employing multiple catheters and source dwell positions. They need to be taken into account, however, in the development of commissioning procedures for TPS dosimetric algorithms.

The TG186 has proposed a graded approach for the commissioning of advanced dose calculation algorithms beyond TG43.^(8)^ Level I commissioning procedures involve the verification of the advanced dosimetry algorithm's ability to reproduce consensus TG43 data^(31)^ for sources supported by the TPS. Uncertainties in the TG43 algorithm of the TPS (Figs. 2 and 3) or the optimization criteria of advanced dose calculation algorithms (Fig. 6), could lead to significant differences in bounded, single‐source geometries and hinder the establishment of definitive acceptance criteria. Single‐source tests are the hardest ones for any advanced dosimetry algorithm; the geometry factor combined with the use of multiple‐source dwell positions in clinical dosimetry planning could render some differences clinically insignificant, and advanced algorithm settings are optimized for optimum speed versus accuracy in clinical brachytherapy. This makes the recommendation to carefully examine differences and to understand and document their clinical impact^(8)^ more useful than the 2% dose difference criterion adopted from TG43U1.^(32)^

Level II commissioning refers to checking the algorithm's efficiency in accounting for heterogeneities and scatter conditions using reference, MC‐generated or experimentally determined dose distributions for particular test geometries.^(8)^ A working group has been formed to address limited test case availability for following the workflow proposed by TG186. A theoretical source has been prepared that has been included in test systems of both vendors of TPSs incorporating advanced dosimetry algorithms.^(33)^ Example test case plans and reference results are being developed^(34)^ that will be made available through a web registry. The aim is that these test cases will be uniform amongst vendors and included in their commissioning test procedures to depict differences between TG43 and advanced algorithms.^(8)^ The independent development of test cases and their sharing through the above‐mentioned registry, after review, is also encouraged.^(8)^ It is envisioned that BrachyGuide can serve as a valuable tool in this process, as well as in retrospective patient cohort studies of dosimetry planning.

## V. CONCLUSIONS

BrachyGuide, a brachytherapy dedicated software tool developed to expedite the configuration of MCNP input files for plans exported in DICOM RT, featuring also a graphical user interface and a DICOM RT viewer, was presented. It was benchmarked through the comparison of MC calculations performed using BrachyGuide‐generated input files to corresponding results from two commercially available TPSs. These comparisons showed TPS, algorithm, and source‐specific differences greater than MC type A uncertainties that were explained by TPS‐related issues and other sources of type B uncertainty. These differences have to be taken into account in commissioning procedures of brachytherapy TPS dosimetry algorithms.

## ACKNOWLEDGMENTS

Research cofinanced by the European Union (European Social Fund–ESF) and Greek national funds through the Operational Program “Education and Lifelong Learning Investing in Knowledge Society” of the National Strategic Reference Framework (NSRF). Research Funding Program: Aristeia. Varian Medical Systems (Palo Alto, CA) and Nucletron, an Elekta company (Veenendaal, The Netherlands) are gratefully acknowledged for providing Brachyvision Acuros v. 10.0.33 and Oncentra Brachy v4.4 for research purposes.
